# Acupuncture for diminished ovarian reserve: Protocol for a systematic review and meta-analysis

**DOI:** 10.1097/MD.0000000000016852

**Published:** 2019-08-23

**Authors:** Ruihong Ma, Jiayi Song, Jinhua Si, Yan Liu, Xinyun Li, Rui Cheng, Zuxian Hu, Tian Xia, Jingbo Zhai

**Affiliations:** a Reproductive Center, First Teaching Hospital of Tianjin University of Traditional Chinese Medicine; b Library, Tianjin University of Traditional Chinese Medicine; c Institute of Traditional Chinese Medicine, Tianjin University of Traditional Chinese Medicine, Tianjin, China.

**Keywords:** acupuncture, diminished ovarian reserve, meta-analysis, protocol, systematic review

## Abstract

**Background:**

The choice of treatments for diminished ovarian reserve (DOR) still remains a challenging clinical problem in reproductive medicine. Acupuncture could be beneficial for patients with DOR. This study aims to systematically investigate the efficacy and safety of the acupuncture for women with DOR.

**Methods:**

We will search electronic databases including PubMed (1946 to present), EMBASE (1974 to present), Cochrane Central Register of Controlled Trials (CENTRAL, all years), Web of Science (1900 to present), Chinese Biomedical Literatures Database (CBM, 1978 to present), China National Knowledge Infrastructure (CNKI, 1979 to present), WANFANG Data (from 2000 to present) to identify potentially eligible studies. Only randomized controlled trials (RCTs) will be included. Experimental interventions include traditional or contemporary acupuncture. Control interventions include no treatment, sham acupuncture, placebo acupuncture, or another active therapy. The primary outcomes include ongoing pregnancy rate and serum anti-Müllerian hormone level. A meta-analysis will be used to estimate a pooled intervention effect if possible.

**Results:**

The results will be published in a peer-reviewed journal.

**Conclusion:**

The findings will provide further evidence for the management of DOR.

**PROSPERO registration number:**

CRD42018114385.

## Abstract 2

Supplemental Digital Content is available in the text

## 1 Introduction

Diminished ovarian reserve (DOR) refers to a decline in the quantity and quality of oocytes.^[1]^ The etiology of DOR includes congenital, medical, or surgical causes; advanced age; and so forth.^[2]^ DOR contributes significantly to the female subfertility.^[3,4]^ It could increase the incidence of the poor ovarian response, decrease the number of retrieved oocytes, embryo implantation rates, and the success rate of in vitro fertilization (IVF).^[3,4]^ According to 2015 American assisted reproductive technology (ART) national summary report, 31% of women seeking the help of ART were diagnosed with DOR.^[2]^ The percentage of ART cycles using fresh nondonor eggs or embryos that resulted in live births is the lowest in patients with DOR.^[2]^ Moreover, the repeated treatment cycles are time consuming and increase medical cost and physiological and psychological burdens.^[5,6]^

Multiple treatments are tried to improve ovarian response in women with DOR.^[4]^ A trial showed that melatonin could increase high-quality oocytes and embryos but not improve other outcomes compared with placebo in women with DOR.^[4]^ A retrospective cohort study showed that the assisted hatching and intracytoplasmic sperm injection (ICSI) could not improve live birth rates (LBRs) in initial ART cycles for women with DOR.^[7]^ A meta-analysis suggested that randomized controlled trials with more large sample size were needed to confirm the efficacy of dehydroepiandrosterone supplementation for women with DOR.^[8]^ Up to now, there are no optimal treatment options for women with DOR.^[9]^ The choice of treatments for DOR still remains a challenging clinical problem in reproductive medicine.^[9,10]^

Recently, acupuncture has gained increased popularity for the management of female infertility in Western countries.^[11]^ Acupuncture could influence female fertility by 4 possible mechanisms including modulating neuroendocrinological factors; increasing blood flow to the uterus and ovaries; modulating cytokines; and reducing stress, anxiety, and depression.^[12]^ Some clinical studies showed that acupuncture could be beneficial for patients with DOR.^[13]^ For example, a prospective observational study showed that the follicle-stimulating hormone (FSH) level in women with DOR was reduced by 8.75 ± 11.13 mIU/mL (*P* = .002) at week 12 after electroacupuncture treatment.^[14]^ A randomized controlled trial showed that acupuncture could reduce FSH, increase antral follicle count, and improve anxiety status compared with wait list control.^[15]^

The previous systematic reviews mainly focused on therapeutic effects of acupuncture in IVF.^[5,16–19]^ The results from these studies were controversial.^[20]^ In this study, we focus on the efficacy and safety of acupuncture for women with DOR. It was not investigated in the previous systematic reviews. Moreover, the potentially eligible studies from China could be missed because no Chinese medical databases were searched in some of previous systematic reviews. And some have not been updated so far. Overall, it still remains inconclusive whether acupuncture is effective and safe for women with DOR.

This study aims to systematically investigate the efficacy and safety of the acupuncture for women with DOR.

## 2 Methods

This protocol adheres to the Preferred Reporting Items for Systematic reviews and Meta-Analysis Protocols (PRISMA-P).^[21]^

### 2.1 Inclusion criteria

#### 2.1.1 Types of studies

Only randomized controlled trials (RCTs) will be included. No language or publication date will be restricted.

#### 2.1.2 Types of participants

We will include women suffering from DOR, regardless of age, race, nationality, number of ART attempts, type of ART [ICSI, IVF, or preimplantation genetic diagnosis (PGD)].^[22,23]^ Participants should be clinically diagnosed with DOR according to clinical guidelines, consensuses, or defined by trialists. The definition of DOR may be various across studies.^[10]^ Only RCTs giving a clear definition of DOR will be considered for inclusion.

#### 2.1.3 Types of interventions

Experimental interventions include traditional or contemporary acupuncture. Traditional acupuncture is defined as needles inserted in classical meridian points.^[24]^ Contemporary acupuncture is defined as needles inserted in nonmeridian or trigger points.^[24]^ Acupuncture can be conducted around the time of transvaginal oocyte retrieval (TVOR), embryo transfer (ET), and so on.^[5,17]^ The frequency, duration, and timing of acupuncture treatment are unrestricted.

Control interventions include no treatment, sham acupuncture, placebo acupuncture, or another active therapy. Sham acupuncture includes treatments that place a needle in a nearby area other than the acupoints or attach electrodes to the skin and give subliminal stimulation.^[25]^ Placebo acupuncture involves attach a needle to the skin surface of the right acupoint without actual penetration of the needles.^[25]^ The following comparisons will be investigated:

1. Acupuncture alone versus no treatment, sham/placebo acupuncture, or another active therapy;
2. Acupuncture plus another active therapy versus another active therapy alone;
3. Acupuncture plus another active therapy versus another active therapy plus sham/placebo acupuncture.

#### 2.1.4 Types of outcome measures

The primary outcomes include ongoing pregnancy rate (OPR) and serum anti-Müllerian hormone level.^[5,26]^ OPR refers to pregnancy beyond 12 weeks of gestation confirmed by fetal heart activity on ultrasound.^[5]^

Secondary outcomes include clinical pregnancy rate (CPR), biochemical pregnancy rate (BPR), LBR, antral follicle count, basal serum FSH level, basal serum estradiol level, the number of oocytes retrieved, fertilization rate, oocyte cleavage rate, high-quality embryos rate, implantation rate, miscarriage rate, the incidence of adverse events, etc. CPR refers to the presence of at least 1 intrauterine gestational sac or fetal heartbeat confirmed by ultrasound 4 to 6 weeks after ET.^[5]^ BPR refers to a positive human chorionic gonadotropin serum or urine test 11 days after ET.^[5]^ LBR refers to a baby born alive after 24 weeks gestation.^[5]^

### 2.2 Search methods for identification of studies

#### 2.2.1 Electronic searches

We will search electronic databases including PubMed (1946 to present), EMBASE (1974 to present), Cochrane Central Register of Controlled Trials (all years), Web of Science (1900 to present), Chinese Biomedical Literatures Database (1978 to present), China National Knowledge Infrastructure (1979 to present), and WANFANG Data (from 2000 to present) to identify potentially eligible studies. The search strategy was developed by a medical information specialist (JS). The search strategy is presented in supplemental digital content 1. No language or publication date will be restricted.

#### 2.2.2 Searching other resources

The World Health Organization International Clinical Trials Registry Platform, Chinese Clinical Trial Registry, and ClinicalTrials.gov will be searched to identify eligible studies. The reference lists of included studies, relevant systematic reviews will also be checked to identify additional eligible studies.

### 2.3 Data collection and analysis

#### 2.3.1 Selection of studies

Two review authors (YL and RC) will independently run electronic searches and identify other resources. Titles and abstracts of identified studies will be checked to remove duplicates. The remaining records will be screened against the inclusion criteria. If necessary, the full texts will be checked to identify eligible studies. The selection process will be summarized using the PRISMA flow diagram. They will resolve disagreements by discussion or with the assistance by the third reviewer (JS).

#### 2.3.2 Data extraction and management

Two review authors (XL and ZH) will independently extract data using a predesigned data extraction form. The following information will be extracted:

1. General information: title, first author, publication year, design, sample size, randomization, allocation concealment, blinding, etc.
2. Participants characteristics: diagnostic criteria, age, race, nationality, etc.
3. Interventions: type of acupuncture and the control, frequency, duration, etc.
4. Outcomes: prespecified primary and secondary outcomes, etc.

Any disagreement will be resolved by discussion or with the assistance by the third reviewer (JS). If necessary, we will contact the authors of included studies for additional data.

#### 2.3.3 Assessment of risk of bias in included studies

The risk of bias in included studies will be independently assessed by 2 review authors (RM and JS) using the Cochrane risk of bias tool.^[27]^ This tool includes 7 domains: random sequence generation, allocation concealment, blinding of participants and personnel, blinding of outcome assessment, incomplete outcome data, selective outcome reporting, and other potential sources of bias.^[27]^ The risk of bias for each domain will be graded as high, low, or unclear. Any disagreement will be resolved by discussion or with the assistance by the third reviewer (JZ).

#### 2.3.4 Measures of treatment effect

The estimated effect will be summarized using risk ratio with 95% confidence interval (CI) for dichotomous outcomes. For the continuous outcomes, we will express the intervention effect using mean difference with 95% CI when the outcome is measured using the same tool across studies. Otherwise, the standardized mean difference with 95% CI will be used.

#### 2.3.5 Dealing with missing data

We will contact authors of included studies to provide a clarification for the missing data or additional data if necessary. The missing data will be not filled in the meta-analysis. Instead, we will assess the potential impact of missing data on the combined effect by the sensitivity analysis.

#### 2.3.6 Assessment of heterogeneity

The statistical heterogeneity will be tested by Chi^2^ test or *I*^2^ statistic value.^[28]^ The heterogeneity is statistically significant if the *P* value of Chi^2^ test is <0.10 or *I*^2^ is >50%.^[27]^ The sources of the heterogeneity will be investigated using subgroup analysis or metaregression.

#### 2.3.7 Assessment of reporting biases

The reporting bias will be assessed using the visual funnel plot when at least 10 studies are included in the meta-analysis.^[27]^ If the asymmetry of the funnel plot is identified, we will explore possible reasons by the subgroup analysis or sensitivity analysis.

#### 2.3.8 Data synthesis

The statistical analysis will be conducted using RevMan 5.3 software. A meta-analysis will be used to estimate a pooled intervention effect when the population, experimental intervention, comparator intervention, and outcome are similar in more than 1 study. If the statistical heterogeneity is not significant, a pooled intervention effect will be estimated using the fixed-effect model. Otherwise, the random-effect model will be used to combine intervention effects in the individual studies. If quantitative synthesis is not appropriate, a narrative description of the results will be provided.

#### 2.3.9 Subgroup analysis and investigation of heterogeneity

The subgroup analyses will be performed based on the following variables when possible. The heterogeneity of intervention effects across subgroups will be investigated by Chi^2^ test. *P* < 0.05 indicates a statistical significance.^[27]^

1. Different diagnostic criteria of DOR;
2. Type, frequency, and duration of acupuncture or comparator interventions;
3. Timing of acupuncture (such as around the time of TVOR and ET);
4. Type of ART (ICSI, IVF, or PGD).

#### 2.3.10 Sensitivity analysis

The robustness of the findings will be tested using the sensitivity analysis based on the following information if possible.

1. Risk of bias: removing studies with high risk of bias;
2. Statistical model: comparing results from the random-effect model with the fixed-effect model;
3. Sample size: removing the study with the smallest or largest sample size in a meta-analysis;
4. Missing data: removing studies in which >20% of patients lost to follow-up.

#### 2.3.11 Summary of findings table

The quality of the evidence for each outcome will be evaluated using the GRADEpro Guideline Development Tool (https://gradepro.org/). The quality of the evidence can be graded very low, low, moderate, or high based on 5 factors (study limitations, imprecision, inconsistency, indirectness, and publication bias). The quality of the evidence will be presented with a summary of findings table.

### 2.4 Amendments

The change, the rationale, and the date of any amendment will be provided in the final report if the protocol is modified.

### 2.5 Ethics and dissemination

No ethical issues are involved because the data used in this study will be extracted from published RCTs. The findings will be published in a peer-reviewed journal.

## 3 Discussion

The choice of treatments for DOR still remains a challenging clinical problem in reproductive medicine. Acupuncture could be beneficial for patients with DOR. This study aims to systematically investigate the efficacy and safety of the acupuncture for women with DOR. The findings will provide further evidence for the management of DOR.

## Author contributions

**Conceptualization:** Tian Xia, Jingbo Zhai, Ruihong Ma, Jiayi Song.

**Data curation:** Jinhua Si.

**Formal analysis:** Yan Liu, Xinyun Li, Rui Cheng, Zuxian Hu.

**Investigation:** Yan Liu, Xinyun Li, Rui Cheng, Zuxian Hu.

**Methodology:** Tian Xia, Jingbo Zhai, Jinhua Si.

**Software:** Ruihong Ma, Jiayi Song.

**Supervision:** Tian Xia, Jingbo Zhai.

**Writing – original draft:** Ruihong Ma, Jiayi Song, Yan Liu, Xinyun Li, Rui Cheng, Zuxian Hu.

**Writing – review and editing:** Tian Xia, Jingbo Zhai.

## Supplementary Material

Supplemental Digital Content
