# Supplementary material for: Acupuncture for diminished ovarian reserve: Protocol for a systematic review and meta-analysis
Source: Medicine (Baltimore). 2019 Aug 23;98(34):e16852. doi: 10.1097/MD.0000000000016852 (PMC6716689; doi:10.1097/MD.0000000000016852)
Supplement: Supplemental Digital Content [file medi-98-e16852-s001.docx]

Appendix 1.

PubMed:

#1"Needles"[Mesh] OR "Acupuncture"[Mesh] OR "Acupuncture Therapy"[Mesh]

#2 Electroacupuncture*[Title/Abstract] OR Meridian*[Title/Abstract] OR Moxibustion[Title/Abstract] OR Moxabustion[Title/Abstract] OR Acupuncture*[Title/Abstract] OR Pharmacoacupuncture*[Title/Abstract] OR Acupotom*[Title/Abstract] OR Needle*[Title/Abstract] OR Ching Lo[Title/Abstract] OR jing luo[Title/Abstract] OR jingluo[Title/Abstract] OR Acupoint*[Title/Abstract]

#3 #1 OR #2

#4 "Ovarian Reserve"[Mesh]

#5 Ovarian Reserves[Title/Abstract] OR diminished ovarian reserve[Title/Abstract] OR poor ovarian response[Title/Abstract] OR DOR[Title/Abstract] OR POR[Title/Abstract] OR decreased ovarian reserve[Title/Abstract] OR ovarian dysfunction[Title/Abstract]

#6 #4 OR #5

#7 #3 AND #6

Embase:

#1 'acupuncture'/exp OR 'needle'/exp

#2 electroacupuncture*:ab,ti ,kw OR meridian*:ab,ti ,kw OR moxibustion:ab,ti OR Moxabustion :ab,ti ,kw OR acupuncture*:ab,ti ,kw OR pharmacoacupuncture*:ab,ti ,kw OR acupotom*:ab,ti ,kw OR needle*:ab,ti ,kw OR Ching Lo:ab,ti ,kw OR jing luo:ab,ti ,kw OR jingluo:ab,ti ,kw OR Acupoint*:ab,ti ,kw #3 #1 OR #2

#4 'ovarian reserve'/exp

#5 'diminished ovarian reserve':ab,ti ,kw OR 'poor ovarian response':ab,ti ,kw OR dor:ab,ti ,kw OR por:ab,ti ,kw OR 'decreased ovarian reserve':ab,ti ,kw OR 'ovarian dysfunction':ab,ti ,kw OR oocyte reserve:ab,ti ,kw

#6 #4 OR #5

#7 #3 AND #6

CENTRAL:

#1 MeSH descriptor: [Needles] explode all trees

#2 MeSH descriptor: [Acupuncture Therapy] explode all trees

#3 electroacupuncture*:ti,ab,kw or meridian*:ti,ab,kw or moxibustion:ti,ab,kw or acupuncture*:ti,ab,kw or pharmacoacupuncture*:ti,ab,kw or acupotom*:ti,ab,kw or needle*:ti,ab,kw OR Ching Lo:ab,ti ,kw OR jing luo:ab,ti ,kw OR jingluo:ab,ti ,kw OR Acupoint*:ab,ti ,kw (Word variations have been searched)

#4 #1 OR#2 OR #3

#5 MeSH descriptor: [Ovarian Reserve] explode all trees

#6 diminished ovarian reserve:ti,ab,kw or poor ovarian response:ti,ab,kw or dor:ti,ab,kw or por:ti,ab,kw or decreased ovarian reserve:ti,ab,kw or ovarian dysfunction:ti,ab,kw (Word variations have been searched)

#7 #5 OR #6

#8 #4 AND #7

CBM:

#1"刺法"[不加权:扩展] OR "针刺补泻"[不加权:扩展] OR "远道刺"[不加权:扩展] OR "扬刺"[不加权:扩展] OR "行针"[不加权:扩展] OR "围刺"[不加权:扩展] OR "透针"[不加权:扩展] OR "齐刺"[不加权:扩展] OR "偶刺"[不加权:扩展] OR "缪刺"[不加权:扩展] OR "毛刺"[不加权:扩展] OR "留针"[不加权:扩展] OR "巨刺"[不加权:扩展] OR "进针"[不加权:扩展] OR "管针"[不加权:扩展] OR "点刺"[不加权:扩展] OR "浮刺"[不加权:扩展] OR "温针疗法"[不加权:扩展] OR "微波针刺"[不加权:扩展] OR "特定组织针刺疗法"[不加权:扩展] OR "神经干刺激疗法"[不加权:扩展] OR "经皮神经电刺激"[不加权:扩展] OR "骨膜针刺术"[不加权:扩展] OR "窦刺"[不加权:扩展] OR "刺淋巴结疗法"[不加权:扩展] OR "特定部位针刺疗法"[不加权:扩展] OR "眼针"[不加权:扩展] OR "头针"[不加权:扩展] OR "手足针"[不加权:扩展] OR "足针"[不加权:扩展] OR "腕踝针"[不加权:扩展] OR "手针"[不加权:扩展] OR "舌针"[不加权:扩展] OR "面针"[不加权:扩展] OR "耳针"[不加权:扩展] OR "唇针"[不加权:扩展] OR "鼻针"[不加权:扩展] OR "手捻针"[不加权:扩展] OR "气针疗法"[不加权:扩展] OR "皮肤针疗法"[不加权:扩展] OR "梅花针疗法"[不加权:扩展] OR "激光针刺"[不加权:扩展] OR "火针疗法"[不加权:扩展] OR "剂量效应关系, 针灸"[不加权:扩展] OR "电针"[不加权:扩展] OR "福尔电针"[不加权:扩展] OR "电热针"[不加权:扩展] OR "电磁针"[不加权:扩展] OR "电刺激疗法"[不加权:扩展] OR "鍉针"[不加权:扩展] OR "声电鍉针"[不加权:扩展] OR "磁鍉针"[不加权:扩展] OR "刺血疗法"[不加权:扩展] OR "长针疗法"[不加权:扩展] OR "超声波针刺"[不加权:扩展] OR "针刺行气"[不加权:扩展] OR "针刺深度"[不加权:扩展] OR "砭法"[不加权:扩展] OR "针刺疗法"[不加权:扩展]

#2"刺法"[常用字段:智能] OR "针刺"[常用字段:智能] OR "远道刺"[常用字段:智能] OR "扬刺"[常用字段:智能] OR "行针"[常用字段:智能] OR "围刺"[常用字段:智能] OR "透针"[常用字段:智能] OR "齐刺"[常用字段:智能] OR "偶刺"[常用字段:智能] OR "缪刺"[常用字段:智能] OR "毛刺"[常用字段:智能] OR "留针"[常用字段:智能] OR "巨刺"[常用字段:智能] OR "进针"[常用字段:智能] OR "管针"[常用字段:智能] OR "点刺"[常用字段:智能] OR "浮刺"[常用字段:智能] OR "温针"[常用字段:智能] OR "经电刺激"[常用字段:智能] OR "窦刺"[常用字段:智能] OR "眼针"[常用字段:智能] OR "头针"[常用字段:智能] OR "足针"[常用字段:智能] OR "腕踝针"[常用字段:智能] OR "手针"[常用字段:智能] OR "舌针"[常用字段:智能] OR "面针"[常用字段:智能] OR "耳针"[常用字段:智能] OR "唇针"[常用字段:智能] OR "鼻针"[常用字段:智能] OR "手捻针"[常用字段:智能] OR "气针"[常用字段:智能] OR "皮肤针"[常用字段:智能] OR "梅花针"[常用字段:智能] OR "火针"[常用字段:智能] OR "电针"[常用字段:智能] OR "电热针"[常用字段:智能] OR "电磁针"[常用字段:智能] OR "电刺激"[常用字段:智能] OR "鍉针"[常用字段:智能] OR "刺血"[常用字段:智能] OR "长针"[常用字段:智能] OR "砭法"[常用字段:智能]

#3 #1 or #2

#4 "卵巢储备功能下降"[常用字段:智能] OR "卵巢储备功能减退"[常用字段:智能] OR "卵巢储备功能降低"[常用字段:智能] OR "卵母细胞质量下降"[常用字段:智能] OR "卵巢低反应"[常用字段:智能] OR "卵巢储备功能低下"[常用字段:智能]

#5 #3 and #4

CNKI:

(SU=刺法 OR SU=针刺 OR SU=远道刺 OR SU=扬刺 OR SU=行针 OR SU=围刺 OR SU=透针 OR SU=齐刺 OR SU=偶刺 OR SU=缪刺 OR SU=毛刺 OR SU=留针 OR SU=巨刺 OR SU=进针 OR SU=管针 OR SU=点刺 OR SU=浮刺 OR SU=温针 OR SU=窦刺 OR SU=眼针 OR SU=头针 OR SU=足针 OR SU=腕踝针 OR SU=手针 OR SU=舌针 OR SU=面针 OR SU=耳针 OR SU=唇针 OR SU=鼻针 OR SU=手捻针 OR SU=气针 OR SU=皮肤针 OR SU=梅花针 OR SU=火针 OR SU=电针 OR SU=电热针 OR SU=电磁针 OR SU=电刺激 OR SU=鍉针 OR SU=刺血 OR SU=长针 OR SU=砭法) AND (SU=卵巢储备功能下降 OR SU=卵巢储备功能减退 OR SU=卵巢储备功能降低 OR SU=卵母细胞质量下降 OR SU=卵巢低反应 OR SU=卵巢储备功能低下)

Wanfang:

(主题:(刺法) + 主题:(针刺) + 主题:(远道刺) + 主题:(扬刺) + 主题:(行针) + 主题:(围刺) + 主题:(透针) + 主题:(齐刺) + 主题:(偶刺) + 主题:(缪刺) + 主题:(毛刺) + 主题:(留针) + 主题:(巨刺) + 主题:(进针) + 主题:(管针) + 主题:(点刺) + 主题:(浮刺) + 主题:(温针) + 主题:(窦刺) + 主题:(眼针) + 主题:(头针) + 主题:(足针) + 主题:(腕踝针) + 主题:(手针) + 主题:(舌针) + 主题:(面针) + 主题:(耳针) + 主题:(唇针) + 主题:(鼻针) + 主题:(手捻针) + 主题:(气针) + 主题:(皮肤针) + 主题:(梅花针) + 主题:(火针) + 主题:(电针) + 主题:(电热针) + 主题:(电磁针) + 主题:(电刺激) + 主题:(鍉针) + 主题:(刺血) + 主题:(长针) + 主题:(砭法)) * (主题:(卵巢储备功能下降) + 主题:(卵巢储备功能减退) + 主题:(卵巢储备功能降低) + 主题:(卵母细胞质量下降) + 主题:(卵巢低反应) + 主题:(卵巢储备功能低下))
